# Underwater Electrochemical Offshore Tests of a Paint Coating Applied in Water on the Legs of an Oil Production Platform

**DOI:** 10.3390/ma17143580

**Published:** 2024-07-19

**Authors:** Juliusz Orlikowski, Krzysztof Żakowski, Michał Szociński, Piotr Igliński, Agata Jażdżewska, Łukasz Gaweł

**Affiliations:** 1 Department of Electrochemistry, Corrosion and Materials Engineering, Faculty of Chemistry, Gdansk University of Technology, 11/12 Gabriela Narutowicza Street, 80-233 Gdansk, Poland; juliusz.orlikowski@pg.edu.pl (J.O.); krzysztof.zakowski@pg.edu.pl (K.Ż.); a.a.jazdzewska@gmail.com (A.J.); lukasz.gawel@pg.edu.pl (Ł.G.); 2 LOTOS Petrobaltic S.A., 9 Stary Dwor Street, 80-758 Gdansk, Poland; piotr.iglinski@lotospetrobaltic.pl

**Keywords:** oil production platform, seawater anti-corrosion protection, underwater coatings, EIS

## Abstract

This paper presents the methodology developed for underwater measurements using electrochemical impedance spectroscopy (EIS) technique, aimed at determining the resistance of an epoxy coating applied in seawater to the legs of an oil production platform. Performing such underwater tests in an offshore environment was technically challenging. The results of measurements obtained on the platform were confronted with comparative results obtained in the laboratory, where the properties of the coating applied in water collected from the Baltic Sea (thickness, hardness, adhesion, and electrical resistance) were examined. This made it possible to conclude about the correctness of the paint coating application by divers on the legs of the platform. The single-layer epoxy coating applied by brush to the platform legs had a resistance above 10 kΩ∙cm^2^ and thus met the assumed minimum resistance of the protective coating cooperating with cathodic protection as the anti-corrosion protection system of the platform legs. The synergy of these two technologies ensures full protection of offshore structures against corrosion. Measurements of the potential of the platform legs confirmed this. Before painting, the potential value at a depth of 0–15 m was 310 ÷ 320 mV versus the zinc reference electrode, while after painting the potential value decreased to 220 ÷ 240 mV, which means that the effect of full cathodic protection was achieved and the platform legs were protected from corrosion. The developed methodology for underwater EIS measurements on the high seas can be applied to any underwater metal structure to assess the quality of protective coatings.

## 1. Introduction

An important part of the world’s energy system is the extraction of oil and gas from beneath the seabed [1]. Petrochemical production platforms have been operating in various parts of the world for more than 60 years [2]. The first ones worked in the Gulf of Mexico in waters no more than 90 m deep [3]. With the development of technology, the largest platforms are now sited in water as deep as more than 300 m.

Control of the rigs’ technical condition and their ability to perform production tasks is carried out through periodic inspections [4,5]. Mobile Offshore Drilling Unit (MODU) jack-up rigs are required to undergo overhaul and classification inspection every five years [6]. The platforms require effective and reliable anti-corrosion protection [7]. Without it, the degradation of corroding material leads to failures or even sometimes disasters [8,9]. The failures are very costly counting directly (the cost of replacing the damaged component and losses due to interruption of production), impose risk to the lives of people working on the facility, and also cause huge indirect costs associated with environmental contamination due to oil spills [10]. The most common anti-corrosion protection for platforms is the application of a paint coating on the metal surface [11,12,13,14]. Renovation of the coating on vessels in the shipyard is a relatively easy task. In the underwater part of the structure, the anti-corrosion protection of the metal consists of the paint coating and, in addition, cathodic protection. These technologies are complementary: the better the barrier properties of the coating, the lower the current density of cathodic protection required to achieve the effect of full protection.

**Figure 1:**
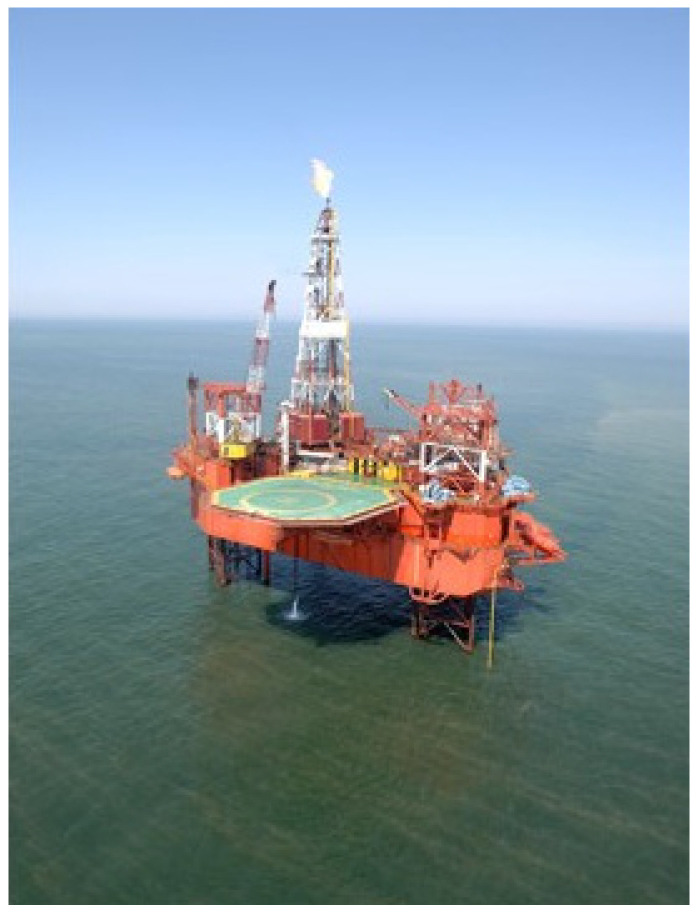
Baltic Beta platform, located on the B3 field on the Polish shelf of the Baltic Sea with the geographical coordinates 55°28′50.67″ N and 18°10′54.03″ E.

In Poland, exploration and exploitation of oil and gas deposits on the Baltic Sea shelf is carried out by the LOTOS Petrobaltic Company. Oil production at the field designated as B3 (about 90 km from land) is performed by the Baltic Beta platform (Figure 1), built around 1980 in France. The platform stands on the seabed on legs submerged in water for about 80 m. On its deck, there are oil processing systems: oil separation, oil pumping to the tanker, seawater preparation for pressing the oil field, and a gas preparation and compression system. While the deposit is under exploitation, the platform is permanently connected to the production wells and the watering wells.

Due to the nature of its operation as a production and extraction unit, the Baltic Beta platform is no longer a mobile unit. This means the inability to leave its location on the seabed. The lack of mobility of the jack-up platform [15] is very important in terms of the anti-corrosion protection system, especially of the platform’s legs. It is impossible to tow the platform to the shipyard and replace the consumed sacrificial anodes or apply a new paint coating system. It is therefore necessary to work on the high seas to ensure effective anti-corrosion protection of the legs, as they undergo intense corrosion, especially in the splash zone [16]. Thus, not in the shipyard, but on the high seas, a retrofit of the cathodic protection system of the platform legs was carried out, placing the systems of sacrificial anodes connected by cable to the platform legs on the seabed [17,18,19]. Also, the application of paint coatings is possible in this case only underwater, which significantly hinders the work. An additional aspect is the evaluation of the effectiveness of coatings applied in this way. The most reliable in this regard are electrochemical tests, in particular the electrochemical impedance spectroscopy (EIS) technique [20,21]. Such tests for evaluating coatings are commonly used in laboratories, but the technique has also been adopted for testing coatings on above-ground industrial steel structures [22]. Performing such a measurement under field conditions requires attaching (gluing) to the coating a measurement cell filled with electrolyte, in which an auxiliary electrode is mounted. The working electrode is the metal substrate covered with the coating under test, i.e., a metal structure. The EIS measurement current flows between the auxiliary electrode and the metal substrate within the measurement cell. However, for the reasons mentioned above, for the Baltic Beta platform, electrochemical measurements must be performed underwater.

The problem of applying paint coating underwater is not new. Work on coating formulations for underwater applications has been going on for a long time. The first coating of this type was released in 1962 by the Shell Chemical Company (Huston, TX, USA) and was used mainly in the water splash zone [23]. In general, all coating systems are based on an epoxy resin binder [24,25]. Over the years, three generations of underwater epoxy that cured in water were developed. The first generation was characterized by high viscosity (the consistency of chewing gum) and degraded quickly (short effective lifetime). The next generation was more durable, but toxic, due to the significant amount of solvent in the paint formulation. The coating was distinguished by its good adhesion to metal surfaces in the water. The current third-generation epoxies are non-toxic, do not crystallize during storage, and, most importantly, are efficient [26,27,28]. At present, there are many types of epoxy paints on the market for use in underwater conditions [29,30,31], and manufacturers assure high product quality and a multi-year warranty under harsh conditions [32,33,34].

In the work presented in this publication, underwater painting of platform legs with epoxy paint was performed. Scientific articles report the use of viscoelastic coatings based on uncrosslinked non-crystalline polymers for painting marine structures as well [35,36]. However, such a coating was not applied in this work due to the authors’ lack of practical experience regarding the application of viscoelastic coating under water.

The application of coatings underwater requires the involvement of a qualified diving team. Before the coating is applied, contaminants and deposits on the structure surface must be removed. Cleaning can be done using abrasives, a water jet, or high-pressure air. Organic coatings are applied underwater with a brush, applicator, or roller. Brush application is the most common. Applying protective coating underwater with a brush is possible when the paint has a low or medium viscosity, which facilitates distribution on the surface of the covered part. The use of a brush makes it possible to paint hard-to-reach areas. In addition, the applied paint evenly penetrates the pores and irregularities of the metal substrate. This method is labor-intensive and inefficient, so it is used to coat small areas.

The works described in this article were undertaken to achieve effective anti-corrosion protection for the legs of the Baltic Beta platform. Protection is achieved by the synergistic action of cathodic protection together with coating protection. The cathodic polarization at a depth of 0–15 m was too low (the protective potential was not reached), which was due to the too low resistance of the old protective coating. As already mentioned, the platform is not mobile, and it is not possible to renew the paint coating in the shipyard. It was therefore necessary to perform the work in the high sea, underwater. Increasing the resistance of the coating cooperating with the cathodic protection makes it possible to increase the cathodic polarization of the structure to the protection potential.

The purpose of the research presented in this paper was to develop guidelines for the technology of underwater renovation of the epoxy coating on the legs of the Baltic Beta platform, as well as to develop a methodology for underwater electrochemical measurements of the applied paint coating. EIS tests carried out underwater were not performed to evaluate the effectiveness of the coating protection as a barrier to isolate metal from the environment. The purpose of the tests was to determine the resistance of the coating applied underwater, which would be sufficient to polarize the legs to a protective potential. 

Conducting the described underwater tests was technically challenging and represents a novelty in the application of the EIS technique to underwater deep-sea surveys performed directly on offshore structures. Determining the optimal resistance value of an epoxy coating applied underwater, while working in combination with a cathodic protection system, is a scientific novelty. The synergy of these two technologies provides full protection of marine structures against corrosion.

## 2. Materials and Methods

### 2.1. Laboratory Research

**Figure 2:**
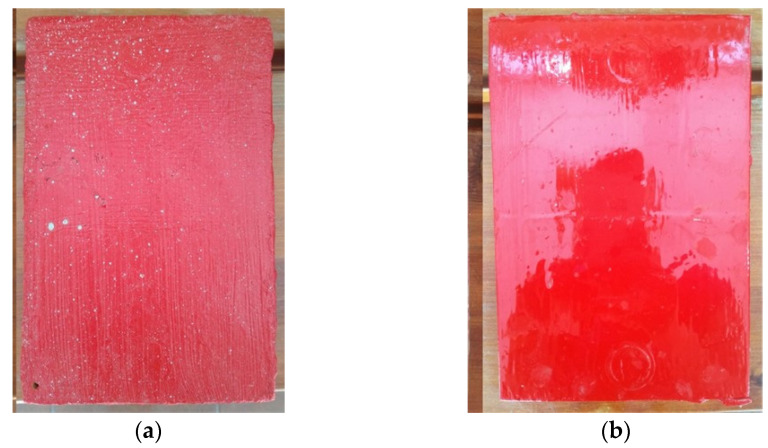
Epoxy-coated steel samples: (**a**) Painting under water; (**b**) Painting in air.

To obtain reference values to compare with, laboratory tests were performed. Two 50 cm × 35 cm plates were prepared from unalloyed structural steel type S235JR with a thickness of 4 mm. They were cleaned by blasting to substrate preparation grade Sa2½. One layer of the epoxy coating designed for application underwater was applied to both plates by brush, but the coating was applied to one plate in water and to the other plate in air. The coating used was Belzona 5831 [37], a two-component epoxy coating that, once cured, provides a barrier to protect structures operated in immersion. Previous experience of the article’s authors with the application of this coating on various structures indicates that it has good adhesion to metallic and non-metallic surfaces. The coating was developed by the manufacturer to be applied underwater. The coating removes water from the surface, ensuring high adhesion to surfaces that are wet when the coating is applied. Application of the coating under the water surface was done in a 0.5-m deep vessel filled with seawater collected from the Baltic Sea. Its basic parameters measured with a Hanna HI 9828 m were as follows: salinity = 6.78‰, resistivity = 84 Ωcm, pH = 8.65, and total dissolved solids TDS = 5970 ppm. After seven days, when the coatings on both plates had cured, a second layer of coating was applied, but only on half of the plates’ surface. A view of both samples after the coatings are cured is shown in Figure 2. 

The following tests were performed on both plates, on the surface covered with one as well as two layers of coating: coating thickness measurement, coating hardness test, pull-off adhesion test, and measurements by EIS technique. The tests were performed at several locations on the surface of the samples to obtain average values of the measured parameters. Large area samples were used, allowing multiple measurements of the same parameter at different locations. Therefore, the number of measurements obtained allows for reliable averaging of parameters and conclusions. The obtained results were used to develop the technology for painting underwater the legs of the Baltic Beta platform, mainly regarding the number of coating layers applied to the fragments of the platform leg selected for painting (the entire surface of the legs was not painted, but only the fragments up to a depth of 15 m with the greatest loss of the original coating).

Measurements of coating thickness, hardness, and adhesion were made in air. The thickness of the coating on the samples was measured using a Phynix Surfix Pro X ultrasonic meter (Phynix Sensortechnik, Neuss, Germany). Hardness measurements were made using a Barcol Hardness Tester Model 934 (Elcometer, Warren, MI, USA). Adhesion of the coating to the substrate was measured by the pull-off method using an Erichsen Adhesion Tester (Erichsen GmbH & Co., Hemer, Germany). EIS measurements in a two-electrode system were made using a Gamry Instruments Reference 600 kit (Gamry Instruments, Warminster, PA, USA) in the frequency range from 100 kHz to 1 Hz. The amplitude of the a.c. perturbation signal was 100 mV. The auxiliary electrode was made of a 4 × 4 cm platinum mesh. The applied frequency range was sufficient to characterize the properties of the applied coating (the high-frequency time constant). With the relatively low magnitude of coating impedance obtained, which implied a lack of coating tightness, extending the measurements towards a lower frequency range would not provide any additional crucial information (the potential presence of low-frequency time constant would just confirm the coating does not prevent from electrochemical reactions on the metal substrate). Additionally, a shorter frequency range allows for faster measurements, which translates to a shorter duration of divers work underwater.

### 2.2. Research on the Oil Production Platform

**Figure 3:**
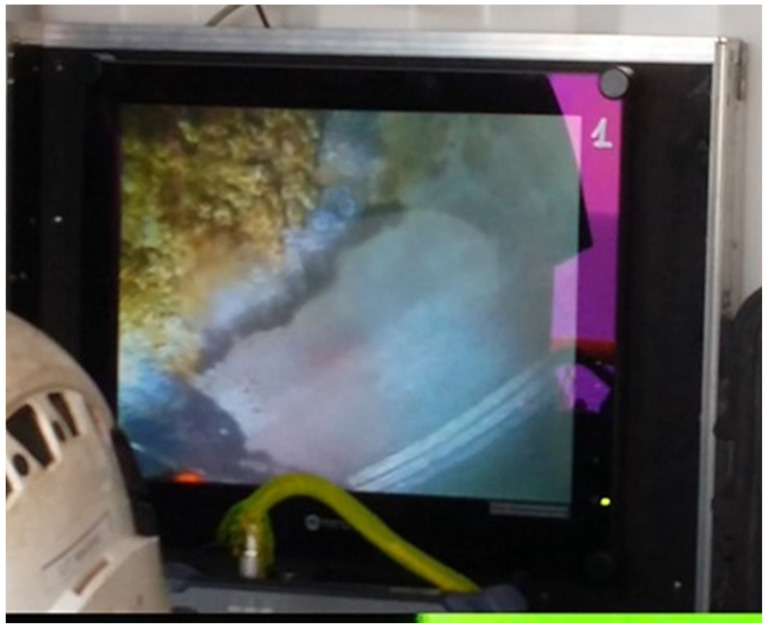
Example of metal surface condition after cleaning before painting.

**Figure 4:**
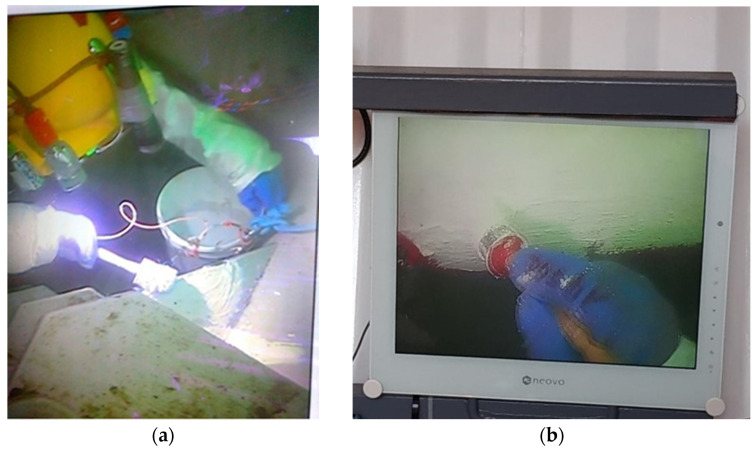
Underwater application of the coating with a brush: (**a**) Painting the pipe connector; (**b**) Painting the pipe.

Cleaning of the platform legs before painting was done with a water jetting method. In areas where the adhesion of the old coating to the substrate was good, the old coating was not peeled off, and cleaning the surface to a metallic state was abandoned. In this situation, the application of a new coating involved obtaining sufficiently good adhesion to the substrate. An example of the surface condition of the structure after cleaning is shown in Figure 3—an image from the camera of a remotely operated underwater vehicle (ROV). The application of the coating by a diver using a brush is shown in Figure 4.

**Figure 5:**
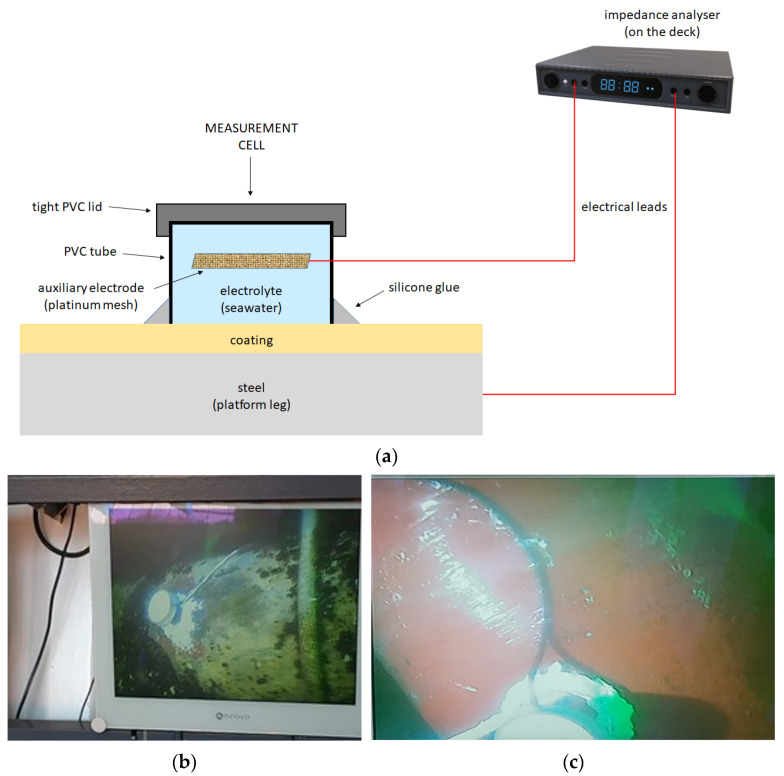
Measurement cells glued to the tested paint coating on the leg of the platform: (**a**) Scheme of the measuring system; (**b**) Old coating; (**c**) New coating (measurement conducted six months after application).

Underwater impedance measurements were made using a PalmSens4 portable impedance analyzer. The measurement cell was made of a PVC tube with a diameter of 7 cm and a height of 5 cm, with a PVC lid glued to it. A platinum mesh auxiliary electrode with dimensions of 4 × 4 cm was placed in the cell, with an electrical lead connecting the auxiliary electrode to the analyzer, operated by personnel on the ship deck (tug boat). The measurement cell was glued to the tested coating with silicone glue (Figure 5), filled with seawater, and sealed. Firm sealing of the cell is required and is particularly important to ensure that the measurement current flows only inside the cell. Underwater measurements were made on the old coating, on the new coating applied to the cleaned steel, and on the new coating applied to the old coating.

## 3. Results and Discussion

### 3.1. Laboratory Research

**Figure 6:**
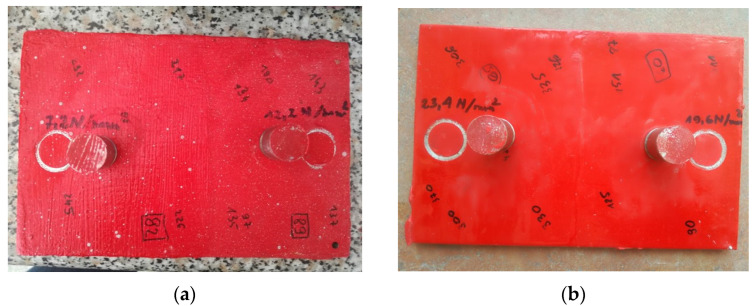
Samples after pull-off adhesion tests: (**a**) Coating applied in water; (**b**) Coating applied in air.

The pull-off adhesion measurement allows for determining the strength of individual coating layers against the force that causes them to peel from the substrate. Figure 6 shows the condition of the samples after one pull-off test. Table 1 shows the average values and deviation of thickness, hardness, and adhesion measurements of the coatings applied to the samples under and over the waterline, depending on the number of layers applied. The values of hardness and adhesion were obtained from 3 measurements, and thickness from 10 measurements.

The target coating thickness reported in the product data-sheet is in the range of 200–400 µm [37]. Values within this range were obtained after applying two layers, both in water and air (Table 1). Comparing the two versions of coating application, it can be seen that the thicknesses of the single layers are similar, while after applying the second layer, a thicker coating was obtained by painting the sample in the air.

The hardness of coatings applied under and over the waterline is not significantly different, indicating that the curing process in both conditions was successful.

Regarding adhesion testing using the pull-off method, satisfactory results were obtained for both coatings applied above and below the water surface. In all measurements, cohesive detachment of the coatings was observed, as can be seen in Figure 6. The coating applied in water had a lower detachment force than that applied in the air, but the high values of the results obtained indicate good adhesion of the coating to the steel substrate. Generally, in offshore, marine, coastal, and subsea environments, it is assumed that if the detachment force is lower than 5 N/mm^2^, the adhesion of the coating to the substrate is insufficient [38]. Meanwhile, the smallest measurement value was 7.2 N/mm^2^ (Table 1).

**Figure 7:**
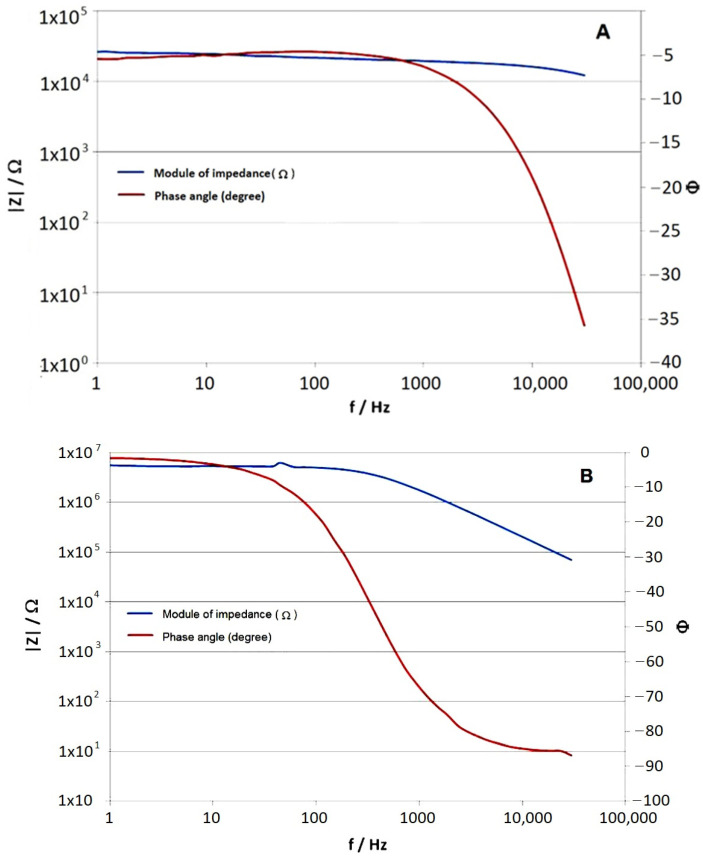
Impedance spectra in Bode format for the coating applied in the water: (**A**) Single layer of coating; (**B**) Two layers of coating.

Figure 7 shows an example of Bode impedance spectra obtained from EIS measurements for a single- and double-layer coating applied underwater, 7 days after coating application (according to the product specification: cure time at 20 °C is 5 days).

**Figure 8:**
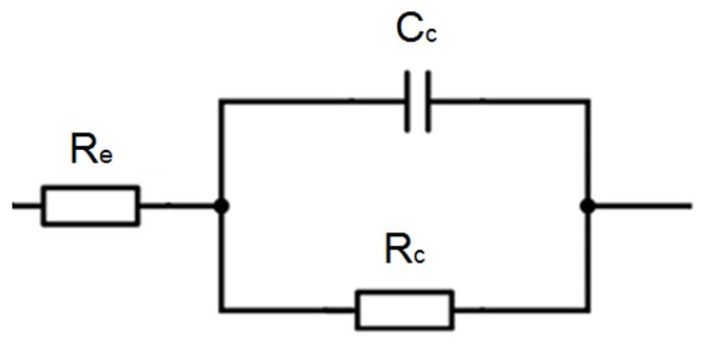
The electrical equivalent circuit is used to analyze the obtained impedance spectra. R_e_—electrolyte resistance, C_c_—coating capacitance, R_c_—coating resistance.

The results presented in Figure 7 show a single time constant in the impedance spectra obtained. Accordingly, an electrical equivalent diagram illustrated in Figure 8 was used to model the impedance spectra [39,40]. Table 2 collects the coating capacitance and resistance values, determined with the ZSimpWin software v3.21. 

In Table 2, it can be seen that the protective properties of the coating applied underwater are significantly lower than those of the coating applied in the air, by up to four orders of magnitude regarding coating resistance. Applying the second layer of epoxy paint increased the resistance of the coating by two orders of magnitude. On the legs of the platform, the epoxy coating cooperates with the cathodic protection system, together providing corrosion protection for the steel. With this in mind, it was assumed that the resistance of the new coating on the platform legs at the level of 10^4^ Ω∙cm^2^ would be sufficient [41,42]. Based on the results obtained, it was therefore considered sufficient to paint the legs of the Baltic Beta platform with a single layer of epoxy coating of ca. 140 μm thickness applied in the water. Not having to apply the two-layer coating system was also of economic importance.

### 3.2. Research on the Oil Production Platform

**Figure 9:**
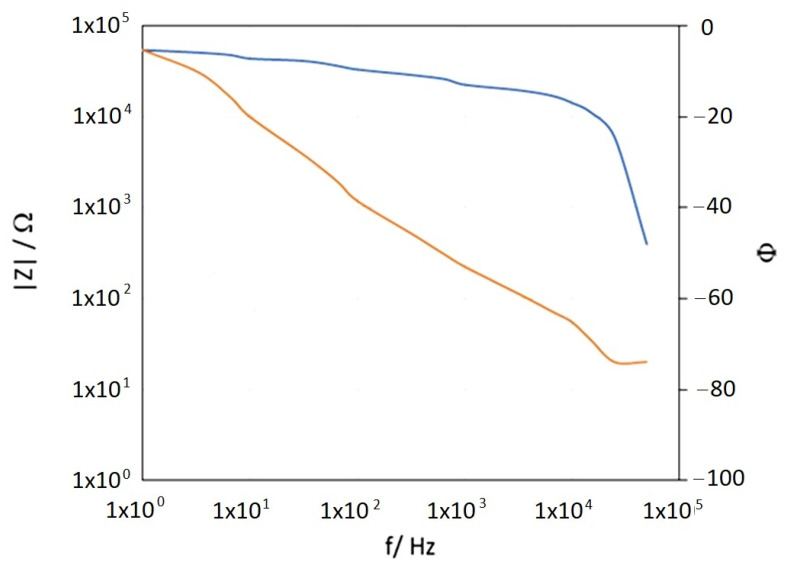
Example of the impedance spectrum collected 6 months after underwater application of a single layer epoxy coating on the platform leg (blue line—impedance modulus, yellow line—phase angle).

An example of the impedance spectrum obtained during the measurement of the new coating applied to the platform leg six months earlier is shown in Figure 9. The results of the impedance tests performed on the Baltic Beta platform (Figure 9) are in good agreement with the values obtained in the laboratory (Figure 7A). Therefore, it can be concluded that the underwater coating application was performed correctly. The obtained resistance of the coating was 55.2 kΩ∙cm^2^, so the assumed value of over 10^4^ Ω∙cm^2^ was achieved.

**Figure 10:**
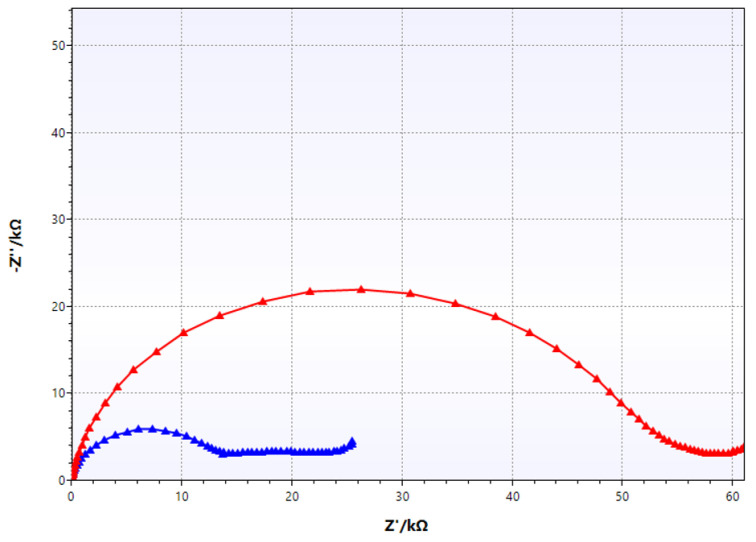
Examples of impedance spectra of a single-layer epoxy coating applied underwater on the platform leg at two locations (red line—depth of 3 m, blue line—depth of 10 m).

Figure 10 presents examples of the Nyquist impedance spectra obtained for the coating applied six months earlier at two locations on the platform legs: at a depth of 3 m (red line) and 10 m (blue line). Analysis of the high-frequency part (the high-frequency semicircle corresponding to coating properties) of the spectra with the ZSimpWin software and the electrical equivalent circuit in Figure 8 showed that the resistance of the coating at a depth of 3 m was 55.2 kΩ∙cm^2^ (red line) compared to a value of 14.6 kΩ∙cm^2^ (blue line) at a depth of 10 m (red line). So, at the first location, the coating had better anti-corrosion properties than at the second one, but at both locations, the coating met the assumed values of its resistance (i.e., more than 10 kΩ∙cm^2^). Thus, it can be concluded that the application of the coating in water was performed correctly, and the assumed resistance of the coating was achieved.

Confirmation of obtaining the desired resistance of the applied coating was provided by the results of measurements of the effectiveness of cathodic protection of the legs. It is implemented using aluminum sacrificial anodes [17]. Before the scheduled painting of the legs, their potential from the water surface to a depth of 15 m ranged from 310 mV to 320 mV versus the zinc reference electrode. According to the criteria given in EN 12473:2014-04 “General principles of cathodic protection in seawater” [43], this means achieving the effect of partial cathodic protection, even though new galvanic anodes have been installed (potential measurements of cathodic protection effectiveness are not the subject of this article). After painting the legs in the section to a depth of 15 m, the potential of the legs decreased and was in the range of 220 ÷ 240 mV. This means achieving a potential criterion for cathodic protection, which, according to EN 12473, is 250 mV vs. zinc reference electrode. The cathodic polarization to the required level was made possible by the synergistic interaction of coating and cathodic protection, which was the goal of the activities described in this work. 

## 4. Conclusions

Based on the conducted analyses (evaluation of the protective properties of the epoxy coating applied in water, the technical feasibility of applying coatings underwater in off-shore conditions, the synergic cooperation of the protective coating with the cathodic protection system, and economic analysis) it was concluded that a single layer of epoxy coating would be applied to the legs of the Baltic Beta platform at a depth of 15 m below waterline.

Renovation of the epoxy protective coating, performed underwater on high seas directly on the platform’s legs, resulted in an improvement in the effectiveness of the working cathodic protection system, which was the main objective of the activities carried out. Applying even a single layer of coating with a brush was enough to reduce the potential of the legs from 310 mV to 220 mV vs. the zinc reference electrode. This ensured that effective corrosion protection of the platform’s legs was achieved, as the potential criterion for cathodic protection (i.e., a potential below 250 mV) was met.

The electrical resistance of a single-layer epoxy coating applied by brush in seawater from the Baltic Sea is of the order of 10^4^ Ω∙cm^2^, while that of a coating applied in air is of the order of 10^8^ Ω∙cm^2^. The coating detachment force is nearly 3 times lower and is about 7 and 20 N/mm^2^, respectively. Although the parameters obtained when the coating is applied in the air are better, the underwater application ensures that the assumed level of interaction with cathodic protection is achieved.

Despite significant technical and implementation challenges on the high seas, it is possible to adapt elements of the EIS apparatus (especially the measurement cell) to perform underwater electrochemical measurements aimed at determining the properties of protective coatings on underwater offshore structures. Such tests were successfully performed on the legs of the Baltic Beta platform.

The developed methodology for underwater EIS measurements can be used on any underwater metal structure to assess the quality of protective coatings. The biggest difficulty is to make a tight measurement cell and attach it to the surface of an underwater structure. Since the measurements are carried out from the deck of the platform (at a distance from the measurement cell), it is also necessary to coordinate the work of divers and measuring personnel.

## Figures and Tables

**Table 1:** Results of coating thickness, hardness, and adhesion measurements.

Coating Application Conditions	Number of Coating Layers Applied	Average Coating Thickness [µm]	Coating Hardness [° Barcol]	Coating Detachment Force [N/mm^2^]
in water	1	139 ± 18	48 ± 3	7.2 ± 0.5
	2	229 ± 7	57 ± 3	12.2 ± 1.2
in air	1	116 ± 17	36 ± 4	19.6 ± 0.9
	2	304 ± 32	65 ± 2	23.4 ± 1.4

**Table 2:** Values of capacitance and resistance of coatings depending on the application conditions.

Coating Application Conditions	Number of Coating Layers Applied	Coating Capacitance [F/cm^2^]	Coating Resistance [Ω∙cm^2^]
in water	1	9.1 · 10^−10^	1.4 · 10^4^
	2	8.1 · 10^−11^	5.2 · 10^6^
in air	1	2.2 · 10^−10^	7.0 · 10^8^
	2	4.9 · 10^−11^	2.1 · 10^10^

## Data Availability

The research has been conducted in cooperation with an industrial partner.
